# Gynecologic and Obstetric Pathologies from Birth to Menopause: Unveiling the Journey and Charting the Future

**DOI:** 10.3390/jcm13206230

**Published:** 2024-10-18

**Authors:** Panagiotis Christopoulos, Ermioni Tsarna

**Affiliations:** Second Department of Obstetrics and Gynecology, School of Medicine, ‘Aretaieion’ University Hospital, National and Kapodistrian University of Athens, 11528 Athens, Greece

Gynecologic and obstetric pathologies encompass a vast array of conditions that affect women’s health throughout their lives. This Special Issue of the *Journal of Clinical Medicine* has served as a platform to explore these complexities, delving into pathologies from the delicate beginnings of life to the hormonal transitions of menopause. As we prepare to conclude this exploration, it is timely to reflect on the recent advancements in the field, acknowledge the knowledge gaps that persist, and illuminate the exciting avenues for future research.

Adolescence, the unique phase that bridges childhood and adulthood, may be accompanied by reproductive complaints and gynecologic pathologies. Obstructive developmental disorders of the reproductive tract, although rooted in intrauterine life, are usually diagnosed during this period, due to primary amenorrhea or severe and worsening dysmenorrhea [1]. Nonetheless, the presentation of complex female genital malformations may be far more challenging, as is their management [2]. A case of obstructed hemivagina with ipsilateral renal agenesis (OHVIRA) has been reported in this Special Issue and challenges regarding presentation and management were extensively discussed (Contribution 8). After menarche, quality of life of adolescent girls may be heavily affected by disorders of menstruation. In particular, heavy menstrual bleeding has been linked with negative perceptions, reduced health related quality of life, and limitations in social and professional life throughout women’s reproductive life [3]. Particularly in adolescence, heavy menstrual bleeding is often the first clinical presentation of primary hemostasis disorders. Given the rarity of some of these disorders, diagnosis and treatment of heavy menstrual bleeding in this setting may be challenging, and gaining a greater understanding of the pathophysiology, epidemiology, and clinical phenotypes of heavy menstrual bleeding can aid to this end (Contribution 4). During adolescence, breast development also takes place, as both glandular elements and adipose tissue grow in response to the steroid hormones [4]. Breast masses are uncommon during adolescence, but affect the patients both physically and psychologically [4]. In this Special Issue, presentation, differential diagnoses, and management of giant juvenile fibroadenomas have been presented based on the cases that were previously published in the literature (Contribution 1).

Pregnancy represents a remarkable period in a woman’s lifespan. Pathologies during this period can affect not only the pregnant woman, but also her offspring’s health in the long-term. As maternal age at childbirth and incidence of obesity have increased, metabolic disorders such as gestational diabetes mellitus have become more common [5]. Preventive strategies for gestational diabetes mellitus have thus become an active field of research. In this Special Issue, the preventive role of dietary patterns in a population with Mediterranean dietary habits has been explored (Contribution 2). Under the Developmental Origins of Disease and Health (DOHaD) theory, stressors during gestation guide epigenetic changes linked to risk for cardiovascular and metabolic disease later in life, a process known as developmental programming [6,7]. Ongoing research is being conducted on the etiology of fetal growth patterns, resulting in both small and large for gestational age neonates. Based on a systematic review and meta-analysis, chronic stress during pregnancy is linked to low birth weight (Contribution 11). With regard to adipokines’ role, which have been implicated in fetal growth restriction, meta-analysis resulted in statistically non-significant results (Contribution 16). Lastly, an original study published in this Special Issue associated calprotectin levels in amniotic fluid already at the second trimester of pregnancy with large-for-gestational-age fetuses, and the authors attributed this finding to low-grade chronic inflammation and oxidative stress as a result of excessive fat deposition (Contribution 14).

A core part of the WHO definition for reproductive health is having “the capability to reproduce and the freedom to decide if, when and how often to do so” [8]. Infertility is defined as failure to conceive after 12 months of regular and unprotected sexual intercourse and is thought to affect 8–12% of reproductive-aged couples, making it one of the most common reproductive pathologies that may affect a woman physically and psychologically [9]. In this Special Issue, the role of Glutathione S-transferase M1 (GSTM1), a member of the detoxification enzymes family, has been explored (Contributions 6 and 9). Homogenous deletion of GSTM1 was linked to female infertility and unfavorable IVF parameters in one study (Contribution 9) and male infertility and unfavorable sperm parameters even in fertile men in another study (Contribution 6). Furthermore, levels of several biomarkers in obese infertile women have been reported along with their association with bariatric surgery and their usefulness as a tool for personalized treatment (Contributions 14 and 7). Even after conception, pregnancy loss represents a common risk that can considerably affect psychological well-being, especially when experienced recurrently. In this Special Issue, a clinically oriented review has presented the current knowledge and advances regarding the etiology, diagnosis, and management of recurrent pregnancy loss (Contribution 3). Lastly, a systematic review examined existing evidence regarding perinatal outcomes in pregnancies achieved with the aid of assisted reproduction techniques and reported an increased incidence of congenital and chromosomal defects, male genital tract defects, and heart defects (Contribution 12). The WHO definition of reproductive health also encompasses family planning and access to modern methods to achieve that which can be influenced not only by availability and accessibility, but also by providers’ views and recommendations [8]. In this Special Issue, a survey that examined the attitude and knowledge of physicians regarding hormonal contraception during breastfeeding has been published (Contribution 7). Furthermore, egg freezing for non-medical reasons with the aim to preserve fertility has been explored (Contribution 13). Social, cultural religious, and economic factors in relation to social egg freezing have been discussed, as well as restricted accessibility based on the woman’s socioeconomic position (Contribution 13).

Sexual health and satisfaction are important aspects of quality of life [10]. Physically and emotionally demanding periods of a woman’s life and pathologies of the reproductive tract can affect sexual life and can therefore contribute to decreased quality of life. In the study of mothers during the early postpartum period published in this Special Issue, it was shown that older, employed women with supporting partners were more satisfied by their sexual life and had greater quality of life (Contribution 10). Another study explored sexuality in postmenopausal women with genital prolapse and concluded that genital prolapse is associated with sexual dysfunction, which should be taken into consideration when management and treatment options are discussed (Contribution 5).

The landscape of gynecologic and obstetric pathologies is constantly evolving, driven by relentless research and technological innovation. This Special Issue has served as a springboard for exploring the complexities of gynecologic and obstetric pathologies across a woman’s lifespan. It has highlighted the commendable progress made in the field while acknowledging the knowledge gaps that persist. As we look towards the future, innovative research focused on the areas outlined above has the potential to revolutionize women’s healthcare. By unraveling the mysteries of these pathologies, we can empower women to lead healthier and happier lives throughout their remarkable journeys.
